# Maximum 11C-methionine PET uptake as a prognostic imaging biomarker for newly diagnosed and untreated astrocytic glioma

**DOI:** 10.1038/s41598-021-04216-5

**Published:** 2022-01-11

**Authors:** Kosuke Nakajo, Takehiro Uda, Toshiyuki Kawashima, Yuzo Terakawa, Kenichi Ishibashi, Naohiro Tsuyuguchi, Yuta Tanoue, Atsufumi Nagahama, Hiroshi Uda, Saya Koh, Tsuyoshi Sasaki, Kenji Ohata, Yonehiro Kanemura, Takeo Goto

**Affiliations:** 1 Department of Neurosurgery, Osaka City University Graduate School of Medicine, 1-4-3 Asahi-machi, Abeno-ku, Osaka, 545-8585 Japan; 2 Department of Neurosurgery, Hokkaido Ohno Memorial Hospital, Hokkaido, Japan; 3 Department of Neurosurgery, Osaka City General Hospital, Osaka, Japan; 4 Department of Neurosurgery, Kinki University Graduate School of Medicine, Osaka, Japan; 5 Departments of Biomedical Research and Innovation, Institute for Clinical Research, National Hospital Organization Osaka National Hospital, Osaka, Japan; 6 Department of Neurosurgery, National Hospital Organization Osaka National Hospital, Osaka, Japan

**Keywords:** CNS cancer, Prognostic markers

## Abstract

This study aimed whether the uptake of amino tracer positron emission tomography (PET) can be used as an additional imaging biomarker to estimate the prognosis of glioma. Participants comprised 56 adult patients with newly diagnosed and untreated World Health Organization (WHO) grade II–IV astrocytic glioma who underwent surgical excision and were evaluated by 11C-methionine PET prior to the surgical excision at Osaka City University Hospital from July 2011 to March 2018. Clinical and imaging studies were retrospectively reviewed based on medical records at our institution. Preoperative Karnofsky Performance Status (KPS) only influenced progression-free survival (hazard ratio [HR] 0.20; 95% confidence interval [CI] 0.10–0.41, *p* < 0.0001), whereas histology (anaplastic astrocytoma: HR 5.30, 95% CI 1.23–22.8, *p* = 0.025; glioblastoma: HR 11.52, 95% CI 2.27–58.47, *p* = 0.0032), preoperative KPS ≥ 80 (HR 0.23, 95% CI 0.09–0.62, *p* = 0.004), maximum lesion-to-contralateral normal brain tissue (LN max) ≥ 4.03 (HR 0.24, 95% CI 0.08–0.71, *p* = 0.01), and isocitrate dehydrogenase *(IDH)* status (HR 14.06, 95% CI 1.81–109.2, *p* = 0.011) were factors influencing overall survival (OS) in multivariate Cox regression. OS was shorter in patients with LN max ≥ 4.03 (29.3 months) than in patients with LN max < 4.03 (not reached; *p* = 0.03). OS differed significantly between patients with *IDH* mutant/LN max < 4.03 and patients with *IDH* mutant/LN max ≥ 4.03. LN max using 11C-methionine PET may be used in prognostic markers for newly identified and untreated WHO grade II–IV astrocytic glioma.

## Introduction

Gliomas are the second most common primary brain tumors according to the 2012–2016 Central Brain Tumor Registry of the United States^1^. Approximately 48.3% of primary malignant brain tumors are glioblastomas, 16.7% are other astrocytomas, and 4.5% are oligodendrogliomas^1^.

Although magnetic resonance imaging (MRI) has been one of the basic and less-invasive imaging modalities used in the management of glioma, brain PET imaging has recently been recommended^2, 3^. We have previously reported a positive correlation between WHO grade and accumulation of 11C-methionine among astrocytomas, but that study did not analyze the relationship with prognosis^4^. Additional analysis was thus performed in the current study. Moreover, the clinical studies investigating the relationship between molecular analysis and uptake of amino acid PET in glioma patients are sparse, and detailed prognostic analyses of associations with molecular profiles and 11C-methionine PET uptake in glioma patients have not been fully completed. This study aimed to evaluate the association between 11C-methionine uptakes, and prognosis in cases of newly diagnosed and untreated adult astrocytic glioma.

## Methods

### Patients

From July 2011 to March 2018, there were 66 adult patients and two patients under 18 years old with newly diagnosed and untreated WHO grade II–IV glioma who underwent surgical tumor resection and preoperative 11C-methionine PET examination, as previously reported^4^. From this previous cohort, we included adult astrocytic glioma patients with *IDH* mutated- *TERT* promoter wild-type, or those with *IDH* wild-type in the present study*.* Finally, a total of 56 patients with astrocytic tumor were included in the present cohort. The 56 patients were comprised of 36 male and 20 female patients, with a mean age of 54.0 years (range, 21–82 years). All 11C-methionine PET was performed within one month prior to tumor resection in glioblastoma patients and within six months in patients with lower-grade glioma. Pathological diagnosis was determined according to the 2016 WHO classification for central nervous system tumors^5^. This study was approved by the institutional review boards at the Graduate School of Medicine, Osaka City University (Approval Numbers: 2047 and 2020-115), and Osaka National Hospital (Approval Number: 0713). Genetic analyses were performed after obtaining written consent. This study was complied with all tenets of the Declaration of Helsinki.

### 11C-methionine PET

An Eminence B PET scanner (Shimadzu, Kyoto, Japan) or Biograph-16 PET scanner (Siemens, Bon, Germany) was used for 11C-methionine PET, according to previously reported procedures^4, 6^. Mean and maximum lesion-to-contralateral normal brain tissue (L/N) ratios were determined by dividing the tumor standardized uptake value by the mean standardized uptake value of the normal contralateral region of the brain, as previously reported^4^.

### Genetic analysis

Genetic analysis was performed as previously described^4^. Genomic DNA was extracted from surgically resected tumor specimens using the DNeasy Blood & Tissue Kit (Qiagen, Valencia, CA, USA) or NucleoSpin Tissue (Machery-Nagel, Duren, Germany). Hotspot mutations of *IDH*1/2 (codon 132 of *IDH*1 and codon 172 of *IDH*2) and *TERT* promoter (termed C228 and C250) were examined using Sanger sequencing with a 3130xLGenetic Analyzer (Thermo Fisher Scientific, Waltham, MA, USA) and Big-Dye® Terminator v1.1 Cycle Sequencing Kit (Thermo Fisher Scientific, Waltham, MA, USA). The methylation status of O^6^-methylguanine-DNA methyltransferase (MGMT) promoter was analyzed using quantitative methylation-specific PCR after bisulfite modification of tumor genomic DNA, as previously reported^7^.

### Survival times

Progression-free survival (PFS) was defined as the time in months between evaluation with 11C-methionine PET and tumor progression according to the Response Assessment in Neuro-oncology working group^8^. Overall survival was defined as the time in months between evaluation with 11C-methionine PET and death.

### Statistical analysis

Patients were subdivided into several groups on the basis of age (≥ 70 or < 70 years), preoperative KPS(≥ 80 or < 80), LN mean(≥ 2.46 or < 2.46), LN max(≥ 4.03 or < 4.03), and extent of resection (biopsy or partial removal, < 90%; subtotal removal, ≥ 90% or gross total removal, ≥ 95%) for statistical analysis.

To compare the patients background characteristics of each group classified according to *IDH* status or LN max or both, we performed statistical analysis using Pearson’s chi-square test. PFS and OS were analyzed using the Kaplan–Meier method. Survival date were evaluated using univariate and multivariate Cox regression analyses. Prognostic factors with a *p* < 0.05 in the univariate analysis were included in the multivariate analysis. The stepwise method was used to evaluate PFS and OS multivariate Cox regression analyses. Statistical significance was defined at the level of *p* < 0.05. All statistical analyses were conducted using EZR software (Saitama Medical Center, Jichi Medical University, Saitama, Japan)^9^.

### Ethical approval

This study was approved by the institutional review boards at the Graduate School of Medicine, Osaka City University (approval numbers: 2047 and 2020-115), and Osaka National Hospital (Approval Number: 0713).

### Consent to participate

Patient informed consents were waived due to the retrospective nature of the study.

### Consent for publication

All authors have approved the manuscript and agree with publication.

## Results

### Patient characteristics

Patient characteristics are summarized in Table 1. Ten patients were classified into *IDH* mutant diffuse astrocytoma, 2 patients with *IDH* mutant anaplastic astrocytoma, 3 patients with *IDH* mutant glioblastoma, 9 patients with *IDH* wild-type diffuse astrocytoma, 10 patients with *IDH* wild-type anaplastic astrocytoma, and 22 patients with *IDH* wild-type glioblastoma. Median LN mean was 2.46 (interquartile range, 1.68–3.04), and median LN max was 4.03 (interquartile range, 2.56–4.89).

**Table 1:** Patient characteristics and histology based on the revised WHO 2016 classification

	Value	Pathology			
		DA	AA	GBM	*P* value
Sex					0.202
Female	20	6	7	7	
Male	36	13	5	18	
Age(years), median (IQR)	59 (40–70)				**0.021**
≥ 70	15	1	4	10	
< 70	41	18	8	15	
Contrast Enhancement in MRI					** < 0.0001**
Yes	42	6	11	25	
No	14	13	1	0	
KPS, median (IQR)	80 (60–100)				** < 0.0001**
≥ 80	35	19	8	8	
< 80	21	0	4	17	
LN mean, median (IQR)	2.46 (1.68–3.04)				** < 0.0001**
≥ 2.46	28	2	7	19	
< 2.46	28	17	5	6	
LN max, median (IQR)	4.03 (2.56–4.89)				** < 0.0001**
≥ 4.03	28	2	6	20	
< 4.03	28	17	6	5	
*IDH* status					**0.00897**
Mutant	15	10	2	3	
Wild-type	41	9	10	22	
*TERT* promoter status					**0.0133**
Mutant	19	2	4	13	
Wild-type	37	17	8	12	
MGMT					0.693
Met	30	11	5	14	
Un-Met	26	8	7	11	
Treatment					**0.00962**
Biopsy	6	2	4	0	
PR	17	6	5	6	
STR, GTR	33	11	3	19	
Adjuvant Therapy					**< 0.0001**
None	18	16	1	1	
CRT	33	2	9	22	
RT Only	2	1	1	0	
Chemo Only	3	0	1	2	
*IDH* status/LN max					**< 0.0001**
Mutant/ < 4.03	12	10	1	1	
Mutant/ ≥ 4.03	3	0	1	2	
Wild-type/ < 4.03	16	7	5	4	
Wild-type/ ≥ 4.03	25	2	5	18	

*IQR* interquartile range, *MRI* magnetic resonance imaging, *KPS* Karnofsky performance status, *LN* lesion-to-contralateral normal brain tissue, *IDH* isocitrate dehydrogenase, *TERT* telomerase reverse transcriptase, *MGMT* O^6^-methylguanine-DNA-methyltransferase, *CRT* chemoradiotherapy, *RT* radiation therapy, *Chemo* chemotherapy, *PR* partial resection, *STR* subtotal resection, *GTR* gross total resection, *DA* diffuse astrocytoma, *AA* anaplastic astrocytoma, *GBM* glioblastoma.

*P* values in bold font are statistically significant.

### Univariate and multivariate analyses for PFS and OS

**Figure 1:**
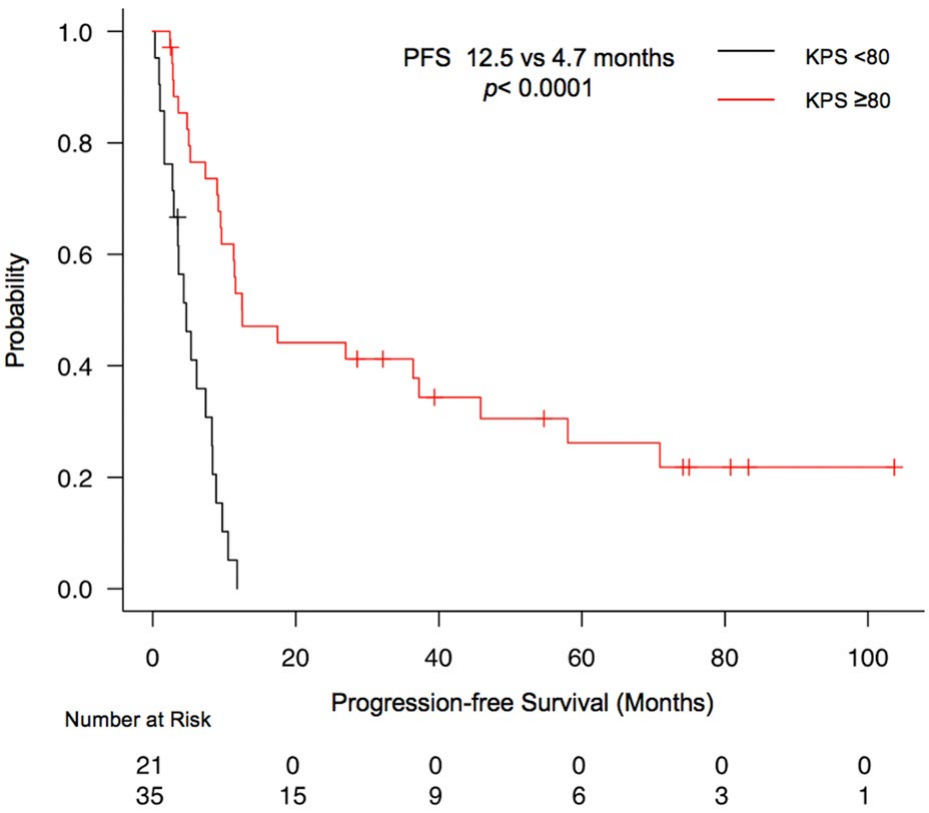
Kaplan–Meier plot of PFS in relation to preoperative KPS.

In univariate analysis, age, enhancement on MRI, preoperative KPS, histology, *IDH* status, and *TERT* promoter status influenced PFS, whereas age, enhancement on MRI, preoperative KPS, LN mean, LN max, histology, adjuvant therapy, and *IDH* status influenced OS (Table 2, Fig. 1). In multivariate Cox regression analysis, preoperative KPS only influenced PFS (HR 0.20, 95% CI 0.1–0.41, *p* < 0.0001), whereas histology (anaplastic astrocytoma: HR 5.3, 95% CI 1.23–22.8, *p* = 0.025; glioblastoma: HR 11.52, 95% CI 2.27–58.47, *p* = 0.0032), preoperative KPS ≥ 80 (HR 0.23,95% CI 0.09–0.62, *p* = 0.004), LN max ≥ 4.03 (HR 0.24, 95% CI 0.08–0.71, *p* = 0.01), and *IDH* status (HR 14.06, 95% CI 1.81–109.2, *p* = 0.011) were influential factors on OS (Table 3).

**Table 2:** Prognostic factors for PFS, and OS in the univariate analyses. *P* values in bold font are statistically significant.

	PFS			OS		
	Time (month)	95% CI	*P* value	Time (month)	95% CI	*P* value
Sex			0.15			0.52
Female	10.5	5.0–45.8		83.3	12.6- Not Reached	
Male	8.3	4.3–11.4		35.9	20.5–56.6	
Age			** < 0.0001**			** < 0.0001**
≥ 70	3.6	1.0–6.1		12.8	5.7–29.3	
< 70	9.7	8.3–36.4		83.3	30.1- Not Reached	
Enhancement in MRI			**0.03**			**0.002**
Yes	8.3	4.7–9.7		27.1	13.3–39.8	
No	37.2	5.3–70.9		Not Reached	52.3- Not Reached	
KPS			** < 0.0001**			** < 0.0001**
≥ 80	12.5	9.2–45.8		83.3	39.8- Not Reached	
< 80	4.7	2.8–8.3		12.6	7.4–27-27.1	
LN mean			0.1			**0.008**
≥ 2.46	6.1	3.5–9.7		26.1	10.4–35.9	
< 2.46	11.8	7.4–37.2		Not Reached	30.1- Not Reached	
LN max			0.19			**0.03**
≥ 4.03	7.3	3.6–10.5		29.3	12.8–39.8	
< 4.03	11.3	5.3–37.2		Not Reached	20.5- Not Reached	
Histology			**0.0003**			** < 0.0001**
DA	37.2	9.5–70.9		Not Reached	52.3- Not Reached	
AA	9.6	5.3–11.8		27.1	11.7- Not Reached	
GBM	4.7	2.9–8.3		20.5	7.7–30.1	
*IDH* status			**0.013**			** < 0.0001**
Mutant	45.8	9.2–70.9		Not Reached	Not Reached- Not Reached	
Wild-type	7.4	4.3–9.7		26.1	12.8–39.8	
*TERT* promoter status			**0.019**			0.054
Mutant	5.4	2.8–9.7		13.3	7.4–56.6	
Wild-type	10.5	7.4–37.2		52.3	26.1- Not Reached	
MGMT			0.77			0.81
Met	9.7	3.0–17.4		52.3	12.8- Not Reached	
Un-Met	8.9	5.4–11.3		27.1	18.3- Not Reached	
Adjuvant Therapy			0.0651			**0.0002**
None	37.2	9.2–70.9		Not Reached	Not Reached- Not Reached	
CRT	7.4	4.7–9.6		26.1	12.8–30.0	
RT Only	9.2	0.9-Not Reached		32.4	12.6- Not Reached	
Chemo Only	1.6	1.0-Not Reached		7.4	5.7- Not Reached	
Treatment			0.69			0.14
Biopsy	5.3	0.9- Not Reached		Not Reached	12.6- Not Reached	
PR	7.4	3.0–12.5		18.3	6.2- Not Reached	
STR, GTR	9.5	7.4–12.5		48.9	29.3- Not Reached	

*MRI* magnetic resonance imaging, *KPS* Karnofsky performance status, *LN* lesion-to-contralateral normal brain tissue, *DA* diffuse astrocytoma, *AA* anaplastic astrocytoma, *GBM* glioblastoma, *IDH* isocitrate dehydrogenase, *TERT* telomerase reverse transcriptase, *MGMT* O^6^-methylguanine-DNA-methyltransferase, *Met* methylation, *CRT* chemoradiotherapy, *RT* radiation therapy, *Chemo* chemotherapy, *PR* partial resection, *STR* subtotal resection, *GTR* gross total resection, *PFS* progression-free survival, *CI* confidence interval, *NA* not applicable, *OS* overall survival.

**Table 3:** Prognostic factors for PFS, and OS in the multivariate analyses

	PFS			OS		
	HR	95% CI	*P* value	HR	95% CI	*P* value
Sex						
Female						
Male						
Age						
≥ 70	Excluded by factor selection with step-wise method			Excluded by factor selection with step-wise method		
< 70						
Enhancement						
Yes	Excluded by factor selection with step-wise method			Excluded by factor selection with step-wise method		
No						
KPS						
≥ 80	0.20	0.1–0.41	** < 0.0001**	0.23	0.09–0.62	**0.004**
< 80	Reference			Reference		
LN mean						
≥ 2.46				Excluded by factor selection with step-wise method		
< 2.46						
LN max						
≥ 4.03				Reference		
< 4.03				0.24	0.08–0.71	**0.01**
Histology						
DA	Excluded by factor selection with step-wise method			Reference		
AA				5.3	1.23–22.8	**0.025**
GBM				11.52	2.27–58.47	**0.0032**
*IDH status*						
Mutant	Excluded by factor selection with step-wise method			Reference		
Wild-type				14.06	1.81–109.2	**0.011**
*TERT* promoter						
Mutant	Excluded by factor selection with step-wise method					
Wild-type						
MGMT						
Met						
Un-Met						
Adjuvant therapy						
None				Excluded by factor selection with step-wise method		
CRT						
RT only						
Chemo only						
Treatment						
Biopsy						
PR						
STR, GTR						

*KPS* Karnofsky performance status, *LN* lesion-to-contralateral normal brain tissue, *DA* diffuse astrocytoma, *AA* anaplastic astrocytoma, *GBM* glioblastoma, *IDH* isocitrate dehydrogenase, *TERT* telomerase reverse transcriptase, *MGMT* O^6^-methylguanine-DNA-methyltransferase, *Met* methylation, *CRT* chemoradiotherapy, *RT* radiation therapy, *Chemo* chemotherapy, *PR* partial resection, *STR* subtotal resection, *GTR* gross total resection, *PFS* progression-free survival, *HR* hazard ratio, *CI* confidence interval, *OS* overall survival.

*P* values in bold font are statistically significant.

**Figure 2:**
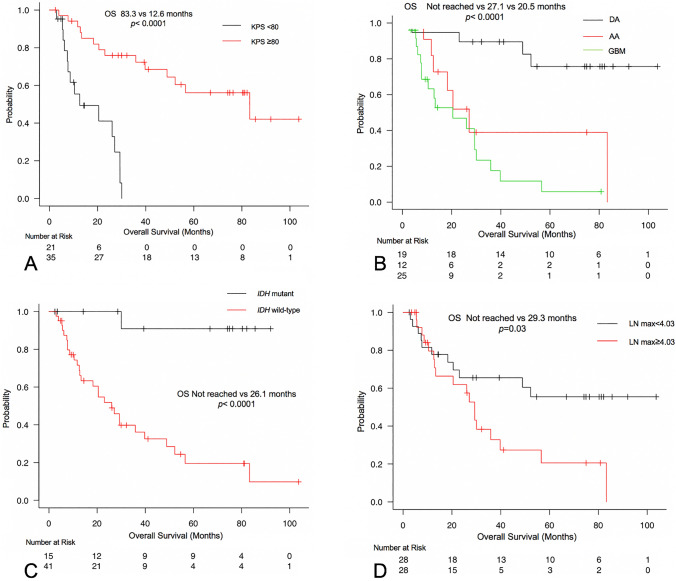
Kaplan–Meier plot of OS in relation to preoperative KPS (**A**), histology (**B**), *IDH* status (**C**), and LN max (**D**).

Median PFS in patients with diffuse astrocytoma, anaplastic astrocytoma, and glioblastoma were 37.2 months, 9.6 months, and 4.7 months, respectively (*p* = 0.0003, Table 2). Median OS was more favorable in patients with preoperative KPS ≥ 80 (83.3 months) than in patients with preoperative KPS < 80 (12.6 months, *p* < 0.0001; Table 2, Fig. 2A). Median OS was not reached for patients with diffuse astrocytoma, 27.1 months for those with anaplastic astrocytoma, and 20.5 months for those with glioblastoma (*p* < 0.0001, Table 2, Fig. 2B). Median OS was more favorable in patients with *IDH* mutation than that in patients with *IDH* wild-type (not reached vs. 26.1 months, respectively, *p* < 0.0001, Fig. 2C). Furthermore, OS appeared shorter in patients with LN max ≥ 4.03 (29.3 months) than in patients with LN max < 4.03 (not reached, *p* = 0.03; Fig. 2D).

### OS in patients classified according to the IDH status/LN max (Fig. 3, Table 2)

**Figure 3:**
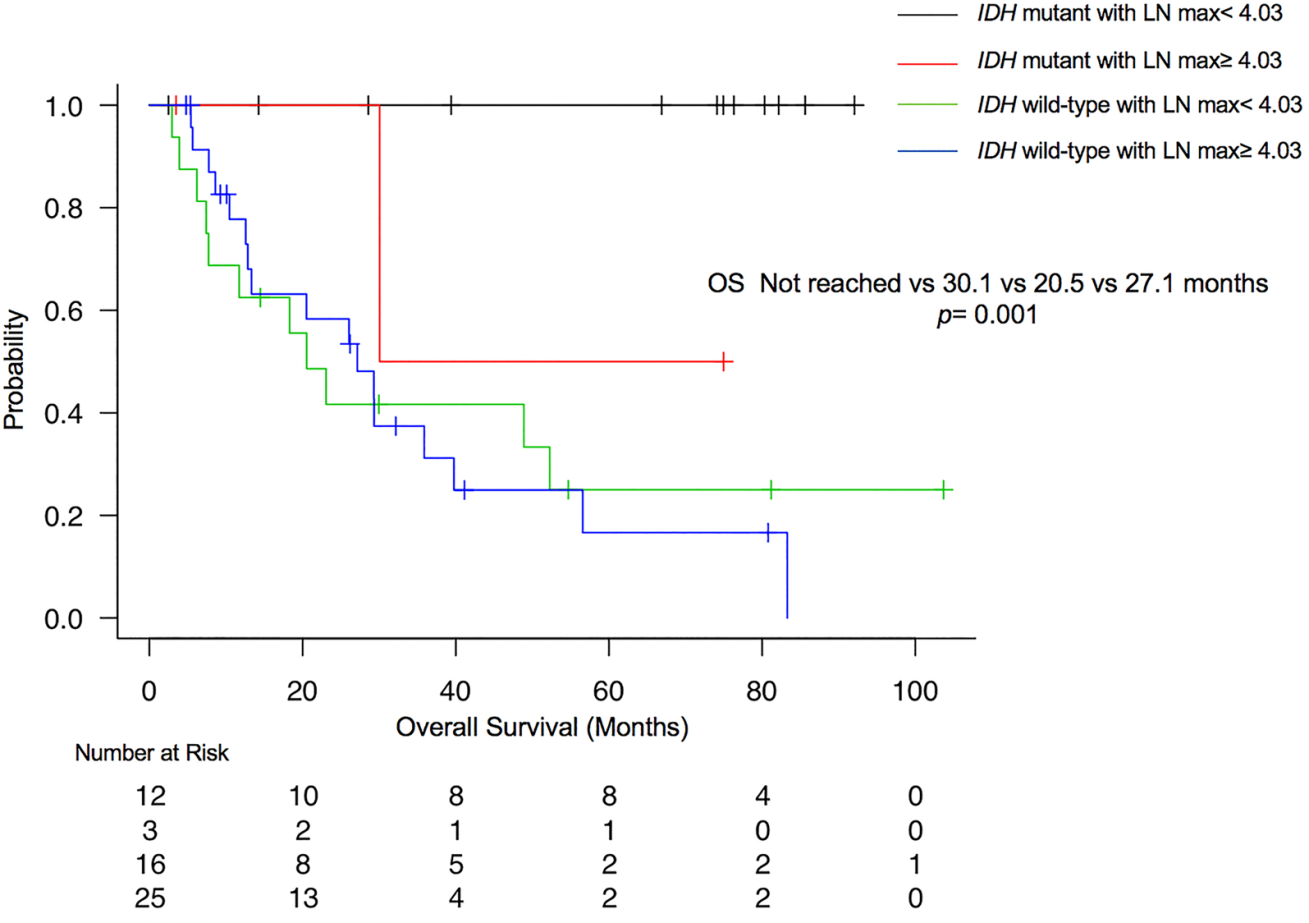
Kaplan–Meier plot of the OS in relation to the *IDH* status/LN max classification. A significant difference in OS existed between patients with *IDH* mutant/LN max < 4.03 and those with *IDH* mutant/LN max ≥ 4.03 (*p* = 0.034), although no significant difference in OS was evident between patients with *IDH* mutant/LN max ≥ 4.03 and those with *IDH* wild-type/LN max < 4.03 (*p* = 0.40), or between patients with *IDH* wild-type/LN max < 4.03 and those with *IDH* wild-type/LN max ≥ 4.03 (*p* = 0.84).

Median OS was not reached for patients with *IDH* mutant/LN max < 4.03, 30.1 (95% CI, 30.1-Not reached) months for those with *IDH* mutant/LN max ≥ 4.03, 20.5 (95% CI, 7.4–52.3) months for those with *IDH* wild-type/LN max < 4.03, and 27.1 (95% CI, 12.6–39.8) months for those with *IDH* wild-type/LN max ≥ 4.03, respectively (*p* = 0.001). A significant difference in OS was seen between patients with *IDH* mutant/LN max < 4.03 and those with *IDH* mutant/LN max ≥ 4.03 (*p* = 0.034), although no significant difference in OS was seen between patients with *IDH* mutant/LN max ≥ 4.03 and those with *IDH* wild-type/LN max < 4.03 (*p* = 0.40), or between patients with *IDH* wild-type/LN max < 4.03 and those with *IDH* wild-type/LN max ≥ 4.03 (*p* = 0.84).

## Discussion

The revised WHO 2016 classification of the central nervous system tumor requires the pathological diagnosis with molecular analysis to reach a diagnosis of glioma^5^. This molecular information has been said to correlate with prognosis, whereas there is still a matter of debate whether imaging biomarkers help estimation of prognosis. Although MRI remains the gold standard for diagnosing glioma, its role in estimating prognosis is limited^10^. On the other hand, 11C-methionine PET using amino tracer might be useful to detect the tumor, predict the grade or genetic status or both^4, 7, 11–13^, and distinguish tumor recurrence from radiation necrosis^14–16^ in glioma patients, although 11C-methionine PET can only be used in limited institutions that have a cyclotron since 11C-methionine has a short half-life about 20 min. However, relatively few reports have investigated the relationship between the uptake of amino tracer using PET and prognosis in glioma. Moreover, reports investigating prognosis of glioma patients in association with molecular analysis and PET in glioma have been limited^17–21^. Thus, our goal in the present study was to determine whether 11C-methionine PET can be used as an additional imaging biomarker of prognosis.

In the present study, we excluded patients with oligodendroglioma, or those with *IDH* mutated- *TERT* promoter mutated, or both because oligodendroglioma is considered to show better prognosis than astrocytoma and is often accompanied by both *IDH* and *TERT* promoter mutations. Although *TERT* promoter mutation is often seen in oligodendroglioma and primary glioblastoma, prognoses differ markedly between oligodendroglioma and glioblastoma^22, 23^. An argument has also been made regarding the association between uptake of 11C-methionine and oligodendroglioma^13, 24–27^. We have previously reported a positive correlation between WHO grade and the accumulation of 11C-methionine among astrocytomas, and a statistically higher uptake of 11C-methionine in oligodendroglioma than in diffuse astrocytoma^4^. In the current study, median PFS was 37.2 months for patients with diffuse astrocytoma, 9.6 months for those with anaplastic astrocytoma, and 4.7 months for those with glioblastoma, respectively. Median OS was not reached for patients with diffuse astrocytoma, 27.1 months for those with anaplastic astrocytoma, and 20.5 months for those with glioblastoma, respectively. Reuss et al. reported that 139 of 152 patients with diffuse astrocytoma diagnosed according to the WHO 2007 classification of the central nervous system tumors showed *IDH* mutant diffuse astrocytoma, whereas more than half of patients with diffuse astrocytoma were *IDH* wild-type in our cohort^28^. Minniti et al. reported that *IDH* mutant anaplastic astrocytoma was found in 56% of their anaplastic astrocytoma patients^29^. OS in patients with *IDH* wild-type was 2.8 years^29^. The relatively shorter PFS and OS of patients with diffuse astrocytoma and anaplastic astrocytoma in the current study were probably attributable to the fact that the present cohort included more patients with *IDH* wild-type astrocytoma than the previous study. On the other hand, Wakabayashi et al. reported that the median OS in patients with glioblastoma who received Stupp’s regimen was 20.3 months^30^, similar to our result in the current study.

Brain PET imaging has recently been recommended for use in addition to MRI in the management of glioma^2, 3^. Takano et al. reported that PFS was worse with LN max ≥ 2.0 than with LN max < 2.0 using 11C-methionine PET among patients with untreated, lower-grade, non-enhancing gliomas^31^. Discrimination of high-grade glioma from low-grade glioma is usually difficult using MRI alone prior to tumor resection in patients with non-enhancing, lower-grade glioma, so we considered whether 11C-methionine PET can be used to predict the prognosis of glioma. However, we could not find significant differences in PFS between astrocytoma patients with LN max ≥ 4.03 and LN max < 4.03 or between those with LN mean ≥ 2.46 and LN mean < 2.46 in the current study.

Recently, some reports have investigated the relationship between prognosis from molecular analysis and uptake of PET using 18F-fluoro-ethyl-tyrosine (18F-FET) PET^17–19, 32^ and 3,4-dihydroxy-6-18F-fluoro-ethyl-L-phenylalanine (18F-FDOPA) PET^33^. Galldiks et al. in a study of photopenic *IDH* mutant gliomas reported that glioma with 18F-FET accumulation below the level of background healthy brain showed unfavorable outcomes, and thus should be treated more actively^18^. The utility of dynamic 18F-FET PET has also been reported^19^. Suchorska et al. reported that longer minimal time-to-peak analysis using 18F-FET PET was associated with a favorable prognosis in *IDH* mutant astrocytomas^19^. A time-to-peak analysis≥ 25 min was associated with longer PFS and OS in patients with *IDH* wild-type high-grade astrocytoma according to Bauer et al.^32^. Kunz et al. reported homogeneous decreases in intratumoral uptake of 18F-FET over time as a factor associated with poor prognosis in non-enhancing glioma^17^. Using continuous measures of 18F-FDOPA PET, Patel et al. reported LN max and age as prognostic factors for OS in WHO grade I–IV gliomas, and that *IDH* or MGMT status did not correlate with uptake of 18F-FDOPA. In this study, we concluded that patients with LN max ≥ 4.03 displayed unfavorable OS compared to patients with LN max < 4.03 among patients with WHO grade II-IV astrocytoma. We also concluded that patients with LN max ≥ 4.03 showed unfavorable OS compared those with LN max < 4.03 among patients with WHO grade II-IV *IDH* mutant astrocytoma, although no significant difference in OS was evident between *IDH* wild-type WHO grade II-IV astrocytoma with LN max ≥ 4.03 and those with LN max < 4.03. Thus, another molecular imaging markers might be needed to estimate prognosis in *IDH* wild-type astrocytoma.

Some limitations need to be considered for the current study. First, the relatively small cohort of the current study might have influenced statistical analyses. For example, *TERT* promoter status did not influence OS in our cohort, although Arita et al. reported the usefulness of *TERT* promoter status in addition to the *IDH* status^34^. Further study with a larger cohort is thus needed to assess the correlation between prognosis and molecular/imaging biomarkers with amino-tracer PET in patients with astrocytoma. Second, we did not take volumetric analyses into consideration in the current study, although some reports have suggested that metabolic tumor volume did not correlate with survival outcomes^17, 19, 32, 33, 35^.

## Conclusion

LN max using 11C-methionine PET offers a markers for estimating OS in patients with grade II-IV astrocytoma. LN max can also be used as a prognostic imaging biomarker to estimate OS in addition to *IDH* status in *IDH*-mutated astrocytoma.

## Data Availability

The date in the current study are available from the corresponding author on reasonable request.

## Abbreviations

PET: Positron emission tomography
WHO: World Health Organization
KPS: Karnofsky Performance Status
PFS: Progression-free survival
HR: Hazard ratio
CI: Confidence interval
TERT: Telomerase reverse transcriptase
LN: Lesion-to-contralateral normal brain tissue
IDH: Isocitrate dehydrogenase
OS: Overall survival
MRI: Magnetic resonance imaging
MGMT: O^6^-methylguanine-DNA methyltransferase
18F-FET: 18F-fluoro-ethyl-tyrosine
18F-FDOPA: 3,4-Dihydroxy-6-18F-fluoro-ethyl-L-phenylalanine
